# The Use of Recently Developed Histochemical Markers for Localizing Neurotoxicant Induced Regional Brain Pathologies

**DOI:** 10.3390/toxins6041453

**Published:** 2014-04-24

**Authors:** Sumit Sarkar, James Raymick, Larry C. Schmued

**Affiliations:** 1 Division of Neurotoxicology, National Center for Toxicological Research/FDA, Jefferson, AR 72079, USA; E-Mail: Larry.Schmued@fda.hhs.gov; 2 Toxicology Pathology Associates, Jefferson, AR 72079, USA; E-Mail: James.Raymick@fda.hhs.gov

**Keywords:** Fluoro-Gold (FG), Amylo-Glo, Fluoro-Gold dextran, Fluoro-Ruby (FR), pericytes, blood vessels, basement membrane, kainic acid, AD, amyloid A-beta

## Abstract

Neuronal and vascular brain components are interrelated morphologically, physiologically and developmentally. Due to this close interrelationship, it is often difficult to understand the cause and effect relationship between neuronal *vs.* vascular dysfunction and pathology. This review will discuss four of the more promising recent developments for detecting vascular pathology, and will compare them with the labeling pattern seen with markers of glial and neuronal pathology; following exposure to well characterized neurotoxicants. To detect the vascular dysfunction in the brain, we recently developed a Fluoro-Turquoise gelatin conjugate (FT-gel), a fluorescent probe that helps to delineate between healthy *vs.* sclerotic vessels. Similarly, we have investigated the potential for Fluoro-Gold to label *in vivo* all the endothelial cells in the brain as they co-localize with RECA, an endothelial cell marker. We have also developed Amylo-Glo, a fluorescent tracer that can detect neurotoxic A-beta aggregates in the brain. In this article, we will discuss the potential use of these novel histochemical markers to study the neurotoxicant induced brain. We will also discuss neurovascular strategies that may offer novel therapeutic opportunities for neurodegenerative disorders.

## 1. Introduction

Vasculature in the brain plays a crucial role in maintaining homeostatic conditions. In normal conditions, the human brain receives around 20% of the cardiac output. It is extremely important that our brain receives blood constantly because if the cerebral blood flow (CBF) stops, the normal function of the brain will be impaired and will soon result in neuronal injury [1]. It is crucial that we have normal neuronal-vascular relationship for proper functioning of the brain. It is known that every neuron in the human brain has its own capillary [2], and the length of capillaries in the human brain is around 400 miles. However, the length of the brain capillaries are reduced in neurodegenerative disorders such as Alzheimer’s disease (AD) [3,4]. Inadequate blood flow to the brain eventually diminishes the transport of energy substrates and nutrients across the blood brain barrier (BBB) and reduces the clearance of potentially neurotoxic substances from the brain including A-beta. 

The blood brain barrier (BBB) is a selective barrier which is located in the brain vasculature and consists of endothelial cells, glial cells with astrocytic end feet, pericytes and basement membranes which together constitute the neurovascular unit (NVU) [5,6,7]. It also acts as a physical barrier because complex tight junctions between adjacent endothelial cells force most of the molecular traffic to take transcellular route across the BBB. Hyperemia that denotes the link between neuronal activity and regional blood flow (CBF) play an important role in neurovascular disease. Neurodegenerative disorders such as in AD, Parkinson’s disease (PD) and Amyotrophic lateral sclerosis (ALS) are associated with microvascular dysfunction and degeneration in the brain, neurovascular disintegration, defective BBB function or vascular elements [7,8,9]. In these neurodegenerative conditions, blood flow to the brain diminishes and subsequently reduces the supply of oxygen, energy and nutrients., Additionally, this kind of deficit could impair the ability of the BBB to clear the toxic molecules that accumulate or are deposited in the non-neuronal cells or neurons. Recently it has been shown that in AD, progressive decline of memory is associated with neurovascular function [10,11], accumulation of neurotoxic A-beta in the blood vessels as well as in the brain parenchyma [12,13]. BBB breakdown has also been shown in the substantia nigra of mice in MPTP (1-methyl-4-phenyl-1,2,3,6-tetrahydropyridine) induced model of PD [14,15,16]. 

In this review we will elaborate how our novel histochemical tracers have been useful to delineate the difference in microvascular morphology following exposures to different neurotoxins.

## 2. Fluorescent Markers for Pericyte Localization in Brain

Pericytes are integral component of neurovascular units of the brain, also called Rouget cells after French scientist Charles Marie Benjamin Rouget [17]. Recently they have gained tremendous attention as they play an important role in BBB stability and anigiogenesis, as pericytes in the brain express α smooth muscle actin (α-SMA) and desmin, two of the most crucial proteins present in smooth muscle cells [18]. Pericytes are located abluminal to the endothelial cells and luminal to the parenchymal astrocytes. They have round nuclei and long processes that encircle the wall of the endothelial cell [19]. Their morphological position in the neurovascular unit or in the BBB makes them ideal candidates for blood flow regulation in the brain [20]. Although a group of authors suggest the role of pericytes in macrophage activity in response to traumatic brain injury [21], others suggest that pericytes may play a role in bacterial infection and other diseases as well [22]. 

Identification of pericytes in the brain has been difficult due to the lack of specific a marker. Several investigators suggest that pericytes express surface antigens such as CD13, α-SMA, desmin, vimentin and NG-2. However, the specificity of the antibodies that were used to localize the pericytes in the brain is controversial since they not only stain pericytes but also stain other surface antigens in the brain. Recent evidence suggest that pericytes do express CD11b [23] which also stains activated microglia following lung injury [24]. 

## 3. Fluorescent Dextrans

In the past, Fluoro-Gold (FG) has been used extensively as a fluorescent retrograde axonal tracer [25]. When used as such it also labels the vesicles, plasma membrane and nucleolus of the retrogradely labeled neurons. However, when conjugated to dextran, it has the potential to label all the vascular pericytes in the brain when injected into the lateral ventricles of the brain [26]. A second fluorescent dextran, TRITC dextran (Fluoro-Ruby; FR) that traditionally has been used as an anterograde neuronal tracer also has the potential to label the vascular brain pericytes when administered into the lateral ventricles. In addition, we have also developed two more fluorescent tracers specifically, FT-dextran and FITC-dextran which display blue and green fluorescent pericytes following injection into the lateral ventricles of the brain. 

**Figure 1:**
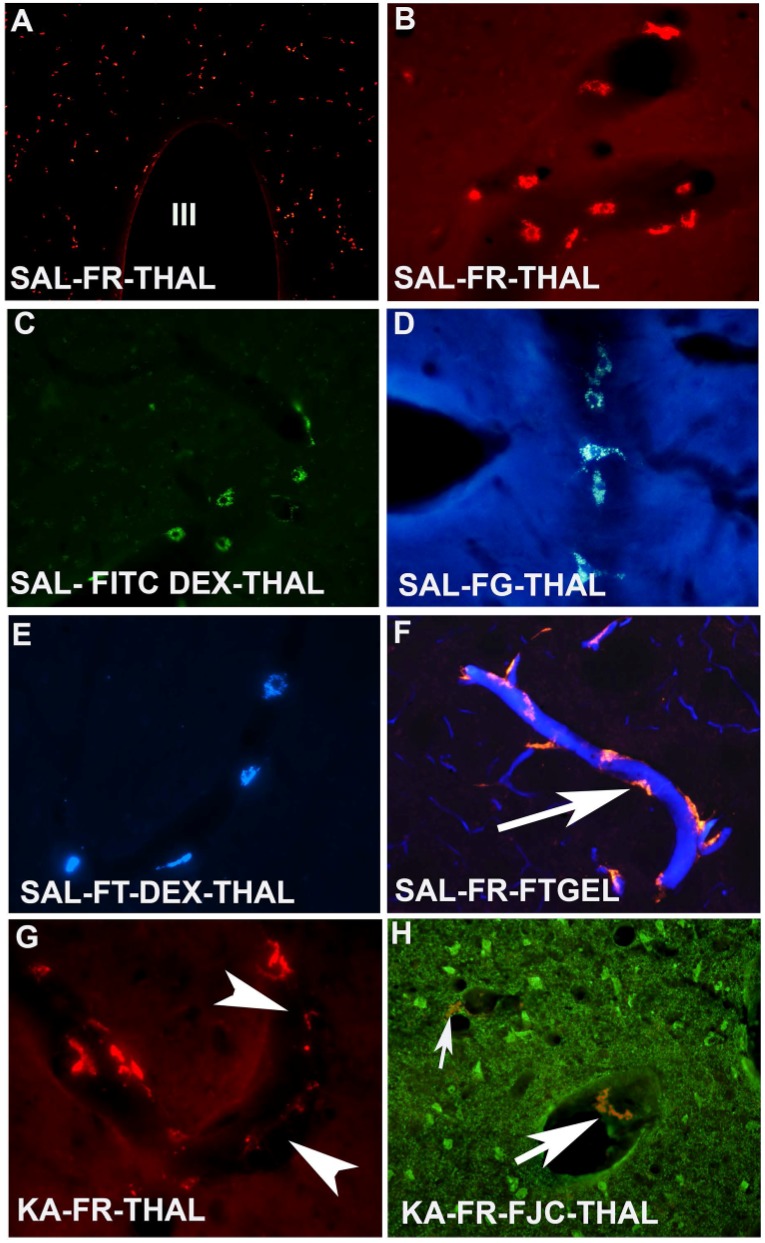
Four different fluorescent dextrans label the brain showing the distribution and morphology of pericytes in the normal and kainic acid treated animals. (**A**) Low power coronal section of the rat brain shows the distribution of Fluoro-Ruby (FR) labeled red color pericytes in the medial diencephalon. III = Third ventricle; (**B**) High power photomicrograph of coronal brain tissue reveals normal pericytes morphology. Note the distinct cellular, lysosomal and nuclear profiles; (**C**) High power coronal section of the brain showing the FITC dextran positive green color pericytes in the thalamus; (**D**) High power photomicrograph of the thalamus showing Fluoro-Gold (FG)-dextran positive yellow bluish colored pericytes; (**E**) High magnification view of normal pericytes labeled with FT-dextran; (**F**) Combined lateral ventricle injection of FR with vascular perfusion of Fluoro-Turquoise gelatin conjugate (FT-gel) shows the abluminal location of pericytes in the normal animal; (**G**) High magnification of faintly FR labeled pericytes displays fragmentation and dysplasia following kainic acid exposure as indicated by arrow heads; (**H**) High magnification photomicrograph of FJC positive degenerating neurons in association with a dysplastic FR labeled pericytes are indicated by arrows.

The procedures to label the pericytes in the brain have been described earlier [26]. Basically, when these fluorochromes were administered into the lateral ventricle of the brain, they have the ability to label perivascular pericytes that surround the capillaries. FITC-dextran shows green fluorescent labeled pericytes (Figure 1C) under blue light excitation while FG-dextran shows yellow blue color and FT-dextran shows blue color under UV excitation (Figure 1D).These four fluorochromes can be used to determine vascular pathology in the brain. FR is the best among all four of them as it is compatible with multiple histochemical procedures as well as immunolabeling (Figure 1A,B). Animals when exposed to kainic acid (KA; an excitotoxin) show extensive neurodegeneration in the thalamus (Figure 1G) and piriform cortex as reported earlier [27]. In the saline treated control animals, pericytes are elongated in shape and distinct cellular, lysosomal and nuclear profiles are evident (Figure 1B). However, following KA exposure, FR labeled pericytes were less in number in the most impacted areas such as the thalamus (Figure 1G), and piriform cortex and the shape of the pericytes also changed significantly. Normally these pericytes encircles the endothelial cells which can be observed by combining FT gel perfusion (Figure 1F). We have also observed that pericytes are detached from the endothelial cells as well as from the basement membrane due to injury from excitotoxicity (Figure 1H). This observation supports the importance of this crucial element of the neurovascular unit in blood flow.

## 4. Fluorescent Dextran Labeling Methodologies

The two fluorescent dextrans, such as Fluoro-Ruby and Fluoro-Turquoise-dextran were obtained from Histo-Chem (Jefferson, AR, USA). FITC-dextran conjugates were prepared by reacting equimolar quantities of FITC (Sigma, St. Louis, MO, USA) with 10,000 m.w. dextran-amine (Invitrogen, Grand Island, NY, USA). Sodium hydroxide was added until the pH stabilized at 9.0. After heating to 60 °C for 1 h, the solution was lyophilized to dryness. The Fluoro-Gold conjugate was prepared by reacting Fluoro-Gold (Histo-Chem, Jefferson, AR, USA) with equimolar quantities of paraformaldehyde (Sigma-Aldrich, St. Louis, MO, USA) and 10,000 m.w. dextran amine (Invitrogen, Grand Island, NY, USA). The solution was heated to 60 °C for 1 h and then dried in a desiccator.

The animals received 1 µL of either FG-dextran, FR, FT-dextran or FITC-dextran in both lateral ventricles. 1–7 days following the i.c.v. injection of the fluorescent tracers, rats were injected i.p. with kainic acid (10 mg/kg), 3-NPA (nitro propionic acid) (30 mg/kg, three doses) or saline. Two days after administration of kainic acid or saline and 6 days after 3-NPA injections, animals were anesthetized with an overdose of Nembutal (100 mg/kg). The animals were first perfused intra-cardially with heparinized saline and then with 10% formalin in neutral phosphate buffer. The brains were then post fixed in 10% formalin plus 20% sucrose for cryoprotection. The brains were sectioned on a sliding freezing microtome at 25 µm thickness and collected in 0.1 M neutral phosphate buffer (PB) and processed for histochemistry. Sections were rinsed three times in distilled water, air dried on a slide warmer for at least 30 min, cleared in xylene and coverslipped with DPX. FG-dextran labeled pericytes appear yellowish in color under UV excitation, whereas FR appeared red under green light excitation, FT-dextran exhibited blue fluorescence under UV excitation and FITC-dextran showed green fluorescence under blue light excitation. 

## 5. Importance and Significance

Recent evidence suggests that the pericyte plays an integral role in maintaining integrity of the BBB as well as in ageing. Our recent study also showed that pericyte loss correlates with neurodegeneration [26]. It is important to note that significant morphological changes such as detachment of pericytes from the blood vessels and endothelium following KA exposure [26] and after 3-NPA injection (presented in this review) were observed. Although the exact cause and effect relationship in these two events is difficult to resolve, direct effect of stimulation of NMDA receptor activation could not be ruled out [28]. It is also possible that the neurodegeneration induced by neurotoxins (KA, 3-NPA) is exacerbated by pericyte mediated vascular changes, BBB breakdown and subsequent hypoperfusion [29]. The above mentioned four novel fluorochrome conjugates are useful to detect and localize the pericytes in the brain in normal and pathological conditions. The association of pericytes with endothelial cells or astrocytes is critical since loss of the contact between them or pericyte dysfunction can lead to development of diseases such as stroke [30], multiple sclerosis [31,32] and brain tumors [33,34]. Since pericytes are key components of the neurovascular units (NVU) of the brain and help mediate vascular function [18,35], understanding the mechanism by which pericytes interact with other components of the NVU as well as other factors that result in dysfunctional pericytes in pathological conditions, are important. The knowledge gained from such studies will help to develop new therapeutic targets in CNS diseases. 

## 6. Vascular Markers in the Brain

There are several methods existing to visualize the vasculature in the brain but few are useful in rodent models, especially in all animal disease models. For example, laminin [36], collagen IV [37] and fibronectin [38] immunohistochemistry have been used to visualize vasculature in the brain. To achieve good immunolabeling, it is often necessary to use antigen retrieval techniques. Collagen IV is a good marker for microvessels for the rat or mice brain, however, Collagen IV, does not show the subtle changes in the brain following neurotoxin exposure. Direct labeling procedures such as DiI, a lipophilic carbocyanin dye [39] has also been used to visualize vasculature in rodents, although this method is not cost effective and is not compatible with immunolabeling or multiple immunolabeling. Therefore, we have tried to develop a method that will enable us to visualize the microvessels in the brain and also be compatible with other histochemical and immunolabeling procedures.

## 7. Fluoro-Turquoise (FT) Conjugate Gelatin (FT-Gel)—A Novel Dye to Visualize the Brain Vasculature and Endothelial Cells

Recently we have developed a new method to visualize the brain vasculature and endothelial cells. The labeling was achieved using Fluoro-Turquoise, a novel blue reactive dye conjugated with gelatin. Very strong blue fluorescence throughout the brain vasculature was seen under UV excitation after vascular perfusion. Due to its unique spectral properties, FT-gel is compatible with numerous common fluorophores, making it ideal for multiple labeling studies. Since the gel has been used to conjugate with FT, the tracer can be permanently fixed within the brain via routine formaldehyde fixation. When perfused with FT-gel, the vascular lumen gets filled with the gel quickly and the abluminal membranes of the endothelial cells lining the blood vessels are rapidly and extensively labeled. We assume that lateral diffusion enables FT-gel to stain membranes that are not in contact with FT-gel solution. FT-gel displays minimal fading upon exposure to UV excitation. The strong signal and low fading characteristics are ideal for serial optical sectioning via laser scanning confocal microscopy, especially at low magnification. 

FT-gel can successfully be delivered via perfusion to the endothelial cell lining of the blood vessels throughout the circulatory system, thus labeling all the blood vessels throughout the entire brain. The intense labeling of FT gel also allows for efficient screening of large areas of the brain at low magnification. When FT-gel labeled sections were double labeled with the endothelial cell marker, RECA-1, all the blood vessels in the brain were co-labeled, thus validating that this novel marker could be used to label both endothelial cells and blood vessels both in normal brains and brains expressing pathologies [40]. Vascular perfusion with FT-gel also led us to show the vascular changes in mice brain following acute, chronic or sub-acute treatment of MPTP [41] further validating the relevance of this unique tracer for microvessel research.

**Figure 2:**
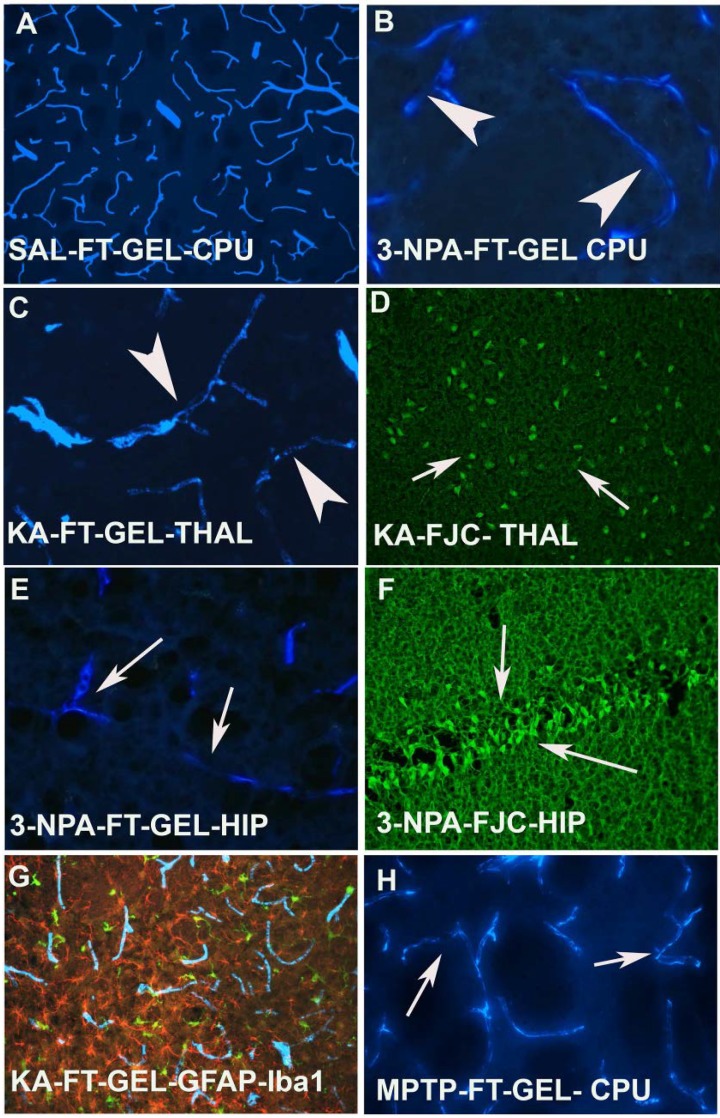
(**A**) High power photomicrograph reveals the distribution of FT-gel labeled blood vessels in the CPU of a saline treated control animal and (**B**) an animal dosed with 3-NPA, where blood vessels are sclerotic (arrow heads), and constricted compared to their saline treated control (A); (**C**) In kainic acid treated animals, not only the number and thickness of microvessels is reduced. The treatment also results in sclerosis, constriction and ischemia (arrow heads); (**D**) FJC labeling confirms degenerating neurons in the thalamus (arrows); (**E**) 3-NPA also affects the hippocampus and resulted in a loss of thickness, ischemia and sclerosis in those microvessels (arrows); (**F**) Sections from the hippocampus of an animal that was dosed with 3-NPA shows significant amount of FJC positive neurons (arrows), thus confirming the concomitant changes with vasculature in the brain; (**G**) Triple labeling demonstrate that FT-gel labeled vasculature (blue) in association with surrounding hypertrophied astrocytes (red) and activated microglia (green) in KA treated rat; combined UV, blue and green light excitation; (**H**) The coronal section of CPU showing the FT gel labeled microvessels following MPTP treatment. Note that the vessels are constricted, sclerotic and ischemic as indicated by arrows.

FT-gel labeled blood vessels are ubiquitously distributed in the brain (Figure 2A), however, when animals were exposed to 3-NPA, a loss of blood vessels within the striatum was obvious. Also within the lesion the blood vessels were sclerotic (Figure 2B). A similar phenomenon was also observed in animals when dosed with KA (Figure 2C). The animals that showed sclerosis and ischemia in the blood vessels also showed neurodegeneration in the thalamus and piriform cortex as reported earlier following KA exposure [27]. 3-NPA injection also resulted in sclerotic, constricted and fainter blood vessels in the hippocampus (Figure 2G) and adjacent sections, when subjected to FJC labeling also showed neurodegeneration (Figure 2F). The FT-gel labeled microvessels can also be combined with the staining of GFAP containing astrocytes and IBA-1 positive microglia (Figure 2F). This combined labeling confirms that both activated microglia and activated astrocytes are in close association with the FT-gel labeled endothelial cells and vascular lumen. Another neurotoxin, MPTP that has been used to induce Parkinsonism in mice also caused the changes in the blood vessels in the striatum (CPU). They appeared sclerotic, fainter in appearance and constricted as compared to saline treated mice (Figure 2H).

## 8. FT-Gel Staining Methodologies

Rats that received either kainic acid or 3-NPA or saline were perfused with FT-gel. Mice that received MPTP were also used in this study. The novel blue fluorescent fluorochrome used is Fluoro-Turquoise, and was obtained from Histo-Chem Inc., Jefferson, AR, USA. Subsequently, 20 g of gelatin was added to 1 liter of physiological saline which was heated up to 65 °C and stirred until the gelatin was fully dissolved. Meanwhile, 150 mg of Fluoro-Turquoise was dissolved in 20 mL of 0.1 M sodium bicarbonate vehicle and slowly added to the gelatin solution while stirring. The temperature was elevated to 80 °C where it was maintained for 10 min, and titrated to pH 9.0 with the 2 M NaOH and then allowed to cool. After cooling to 60 °C, the solution was titrated to pH 7.4 using10% HCl while maintaining the temperature. Two days after kainic acid or saline administration, or 6 days after 3-NPA injection, rats were anaesthetized with an over-dose of pentobarbital (100 mg/kg) and then perfused with 100 ml of heparinized saline followed by 250 mL of 10% formalin fixative in neutral phosphate buffer, followed by a flush with 100 mL of saline and finally with 200–250 mL of FT-gel. MPTP treated mice were also perfused with FT-gel with slight modification as described before [41]. Although the FT-gel solution was maintained at 60 °C prior to perfusion, the temperature of this solution entering the ascending aorta was measured as 55 °C. Brains were subsequently transferred to a 5 °C solution of 20% sucrose dissolved in 10% formalin fixative in 0.1 M neutral phosphate buffer for at least 24 h. Brains were frozen in dry ice and sectioned on a sliding freezing microtome at a thickness of 25 µm and collected in 0.1 M neutral phosphate buffer. Sections were then mounted onto gelatin-coated slides, air dried on a slide warmer, cleared in xylene and coverslipped with DPX (Fluka, St. Louis, MO, USA).

## 9. Importance and Significance

FT-gel perfusion of animals allows us to visualize all brain vasculature. Moreover, FT-gel is compatible with other histochemical labeling methods and also compatible with other fluorophores, making it an ideal marker for multiple labeling studies. Due to its intensely bright fluorescence properties, it is easy to analyze large areas of tissue at relatively low magnification. Since FT-gel can be permanently fixed with formalin, it can be used routinely to demonstrate brain vasculature. FT-gel also co-labeled with endothelial marker RECA-1, confirming it to be an ideal marker of neurovascular unit. When exposed to neurotoxins such as 3-NPA or KA, FT-gel labeled microvessels appear fragmented and ischemic and the same areas show neurodegeneration [Fluoro Jade C (FJC) positive neurons] suggesting the usefulness of this novel marker in the pathological conditions as well.

## 10. Fluoro-Gold (FG)

Although FG was initially introduced as a retrograde tracer to label neurons after focal injection in the brain, the use of this tracer to visualize microvessels/vasculature in the brain was only recently explored. When employed as retrograde tracer, the FG is taken up by neuronal terminals within the injection region [25]. Subsequently, the tracer gets transported retrogradely from the terminals through their axons to the cell bodies of origin and finally into the dendrites of these cells. Although it was reported by Schmued and Fallon in 1986 [25] that FG has the ability to label the vascular cells in the brain, there was no systematic characterization of this tracer in the brain vasculature, it did not address the doses necessary to visualize all the vasculature within the brain, nor did it determine whether FG could be used to detect vascular damage and vascular leakage related to BBB disruption. Recently Bowyer *et al.* [42] have showed that FG could be used to label the vasculature throughout the brain *in vivo* and it could be used to detect vascular structural changes and integrity. FG has also been used to label all the endothelium *in vivo* as all the FG labeled vessels co-localized with RECA-1, an endothelial cell marker. It is also compatible with other fluorescent labeling in histologically prepared brain tissue sections. 

**Figure 3:**
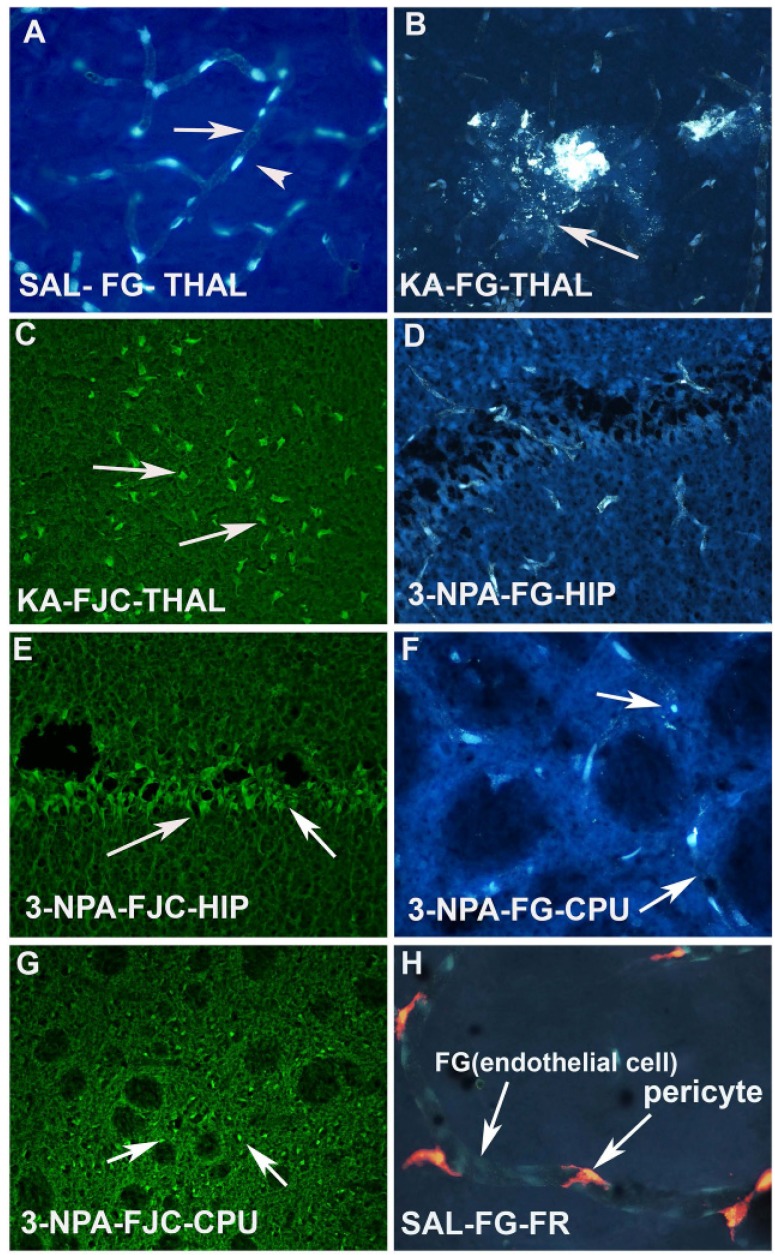
(**A**) Photomicrograph of a coronal section through the thalamus after 20 mg/kg. i.p. injection of F-G in a saline treated control animal. Arrow indicates the labeling of the endothelium and arrow head indicates the position of the nucleus of the endothelial cells; (**B**) High power photomicrograph through thalamus showing the KA-induced damage of the F-G labeled vasculature. Arrow indicates the leakage as well as fragmentation of the vessels due to KA neurotoxin exposure; (**C**) High magnification image through the thalamus showing intensely stained Fluoro Jade C (FJC) positive neurons (arrows) in adjacent brain sections of the same animal that was exposed to KA; (**D**) 3-NPA also resulted damage to the F-G labeled endothelium. Arrow indicates shrinkage and sclerosis of the vessels; (**E**) Adjacent section of the brain of the animal that was exposed to 3-NPA was subjected to FJC labeling in the hippocampus. Arrow indicates the FJC positive degenerating neurons in the hippocampus; (**F**) High power photomicrographs through CPU also shows granulation and fragmentation (arrow) of the endothelium of the vessels following 3-NPA exposure; (**G**) Low power photomicrograph through the CPU of an adjacent section shows FJC positive neurons and neuropil, while myelinated fascicles appear dark (arrows); (**H**) High power photomicrograph shows the association of FG labeled endothelium and FR labeled pericytes in the brain vasculature. Note that FG labeled endothelial cells are yellowish blue while FR labeled pericytes are reddish in appearance.

I.P injection or I.V. injection of FG (20–30mg/kg) labels all the microvessels/endothelial cells of the brain as evidenced in the vehicle treated animal (Figure 3A). However, KA injected animals show leakage of FG, as well as fragmentation of the blood vessels in the thalamus (Figure 3B). The same animals also showed Fluoro Jade C positive degenerating neurons in the thalamus (Figure 3C). Another neurotoxin, 3-NPA also caused sclerosis, fragmentation and ischemic blood vessels in the hippocampus (Figure 3D) and striatum (CPU) (Figure 3F). Both the CPU (Figure 3G) and hippocampus (Figure 3E) also showed FJC positive neurons, thus confirming the correlation of vascular disintegration and neurodegeneration following neurotoxin insult. FG labeled microvessels/endothelial cells are seen to be wrapped by pericytes (Figure 3H). This was observed when animals received injection of FR into the lateral ventricle of the brain. 

## 11. Fluoro-Gold Staining Methodologies

Animals that received kainic acid, 3-NPA or saline were used in this study. Two days after kainic acid injection or 6 days after 3-NPA injection, along with saline treated controls, the animals received FG (20 mg/kg) I.P. 30 min prior to perfusion. To visualize vascular and endothelial cell labeling with FG, animals were given a lethal dose of 150 mg/kg pentobarbital. Rats were perfused with 50 mL of heparinized saline followed by 200 mL of 10% formaldehyde in 0.1M sodium phosphate buffer (pH 7.4). Brains were dissected out from the calvarium and post fixed in the same fixative plus 20% sucrose in 0.1 M sodium phosphate buffer for at least one day. The brain was removed from the sucrose and rapidly frozen in crushed/powdered dry ice. It was then embedded in OCT compound (Electron Microscope Science, Hatfield, PA, USA) and mounted on a Leica Cryostat for cutting coronal sections 25 μm thickness. Subsequently, brain sections were transferred to 5 mM phosphate buffer or distilled water and then mounted on gelatin coated slides. Then slides were dried for 10–30 min at 55 °C on a slide warmer. Slides were then cooled, cleared in xylene and coverslipped with DPX mounting media. FG labeled vasculature and endothelial cells were visible under UV excitation and labeled structures appear golden in color.

## 12. Importance and Significance

FG is a novel tracer that has the potential to label the endothelial cells *in vivo*. FG is compatible with other histochemical as well as immunolabeling procedures. Moreover, it could also detect the vascular leakage following neurotoxic insult that disrupts the BBB integrity. FG could be a very useful tracer to detect vascular changes such as atrophy, increased number of pinocytic vesicles in endothelial cells, increased laminin and collagen content in the basement membrane in AD [43,44,45]. 

## 13. Marker for Amyloid Plaques

One of the salient features of Alzheimer’s disease (AD) pathology is deposition of extracellular amyloid plaques in the forebrain [46]. These plaques were named amyloid since they could be stained with iodine that stains polysaccharides like amylose. Later, it was revealed that this reaction was not specific and plaques primarily contained proteinaceous fibular A-beta aggregates with beta pleated sheet tertiary configuration [47]. Recent evidence suggests that plaques are derived from the trans-membrane amyloid precursor protein (APP), via cleavage of secretase enzymes which generate A-beta _1-40_ and A-beta _1-42_ fragments which in turn aggregate in the form of insoluble plaques. Traditionally, Congo Red [48] and Thioflavin S [49] have been used to localize these plaques in the AD diseased brain. However, both these dyes have limitations as both are excited over a broad range of frequencies that result in “bleed through” a phenomenon when signals can be seen in other filters. This eventually limits their use in the image analysis where one has to acquire images at relatively low magnifications. Other limitations associated with these two dyes are the need to use relatively harsh chemical reagents as well as high dye concentrations to visualize the plaques in the brain.

Immunolabeling methods have also been employed to localize the amyloid plaques components using antibodies directed against A-beta _42_ and A-beta _40_. Although immunolabeling methods have unique advantages over traditional methods of localizing the plaques in the brain, the specificity of these antibodies could be a limitation when the labeling of all portions of the plaques is desired. Immunolabeling procedures are also time consuming and labor intensive. This led us to develop a novel tracer that will not only label the plaques in the brain parenchyma but also vascular plaques in a simple and reliable manner.

## 14. Amylo-Glo

Significant effort was made in the past two decades to develop *in vivo* tracers as PET radioligands for the diagnosis of AD in living patients. Two such classes of amyloid ligands are radio-iodinated benzothiazole and styrylbenzene derivatives [50]. Thioflavin S and Thioflavin T both belong to the benzothiazole family, whereas K114 [51] and BSB [52] are both styrylbenzenes. Although the staining ability of these two tracers is to visualize amyloid plaques in the brain; however, their low resolution and contrast staining and harsh chemical treatment and time consuming histological processes make these two tracers less applicable in daily routine amyloid plaque analysis. Thus, we have investigated the use of styrylbenzene derivative Amylo-Glo to localize the amyloid plaques under mild histological conditions in AD-Tg animals.

### 14.1. Amylo-Glo Staining Methodology

Fixed frozen tissue sections were cut in a freezing microtome or in a cryostat could be used for Amylo-Glo staining. Briefly, tissue sections were mounted onto gelatin coated slides and air dried on a slide warmer at 55–60 °C for 30 min. Subsequently the slides were rinsed in 70% alcohol for 5 min, followed by a 2 min rinse in distilled water (DW). The slides were then incubated in Amylo-Glo solution (Histo-Chem, Jefferson, AR, USA) that was made by diluting 1ml of the 0.1% staining solution with 99 mL of 0.9% saline. The slides were rinsed in saline for 5 min, followed by a brief (15–20 s) rinse in DW. The slides were dried on a slide warmer for 5 min and then coverslipped with DPX mounting medium. Amylo-Glo stained sections could be used for multiple labeling by following normal immunofluorescence methodologies.

### 14.2. Staining Properties

Amylo-Glo stain unequivocally is one of the best fluorescent markers for visualizing amlyloid plaques in the brain in terms of the resolution, contrast, and compatibility with other fluorescent labeling procedures. The resolution obtained by using Congo Red or Thioflavin S is lower than that seen using Amylo-Glo or Pan A-beta _40_ immunofluorescence suggesting that Amylo-Glo has greater affinity for A-beta aggregates. Vascular plaques are also brightly labeled with Amylo-Glo compared to the less intense following Pan A-beta _40_ immunofluorescence. This suggests that there may be structural differences between amyloid A-beta found in the brain and those found in the blood vessels. The spectrophotometric properties are also quite different. While Congo Red is excited by green light and emits red light, Thioflavin S is excited by blue and UV light and emits green light, Amylo-Glo is excited by UV light and emits a bright yellow light. Amylo-Glo appears to have a relatively sharp excitation profile and broad emission profile [53]. Another important aspect of this tracer, its unusual brightness and contrast that attributed to the compounds 5.46-fold increase in fluorescent emission intensity upon binding A- beta aggregates. Similar phenomenon has also been reported for other styrylbenzenes such as K114 and BSB [54]. 

### 14.3. Applications for High Throughput Localization and Image Analysis of Plaque

Amylo-Glo, being an intensely bright fluorescent tracer under UV excitation, can be used for high volume plaque localization and quantification studies. Our previous studies indicate it has twice the brightness of Congo Red or Thioflavin S, when image analysis was done at higher magnification (20×) and 5–6 times brighter when imaged at lower magnification (4×) [53]. It is important to note that at such low magnification neither Congo-Red nor Thioflavin S labeling was visible for image analysis. Thus, Amylo-Glo is more sensitive than other conventional tracers for plaque localization. Another important factor for image analysis is large area quantification. Since large area quantification requires stitching of multiple images at low magnification, this means Amylo-Glo labeling would require less time for tiling and image analysis. Since Congo-Red and Thioflavin S cannot be imaged at 4×, one has to take multiple images at 10×, thus requiring 96 images to be tiled together to visualize one coronal mouse brain section.

**Figure 4:**
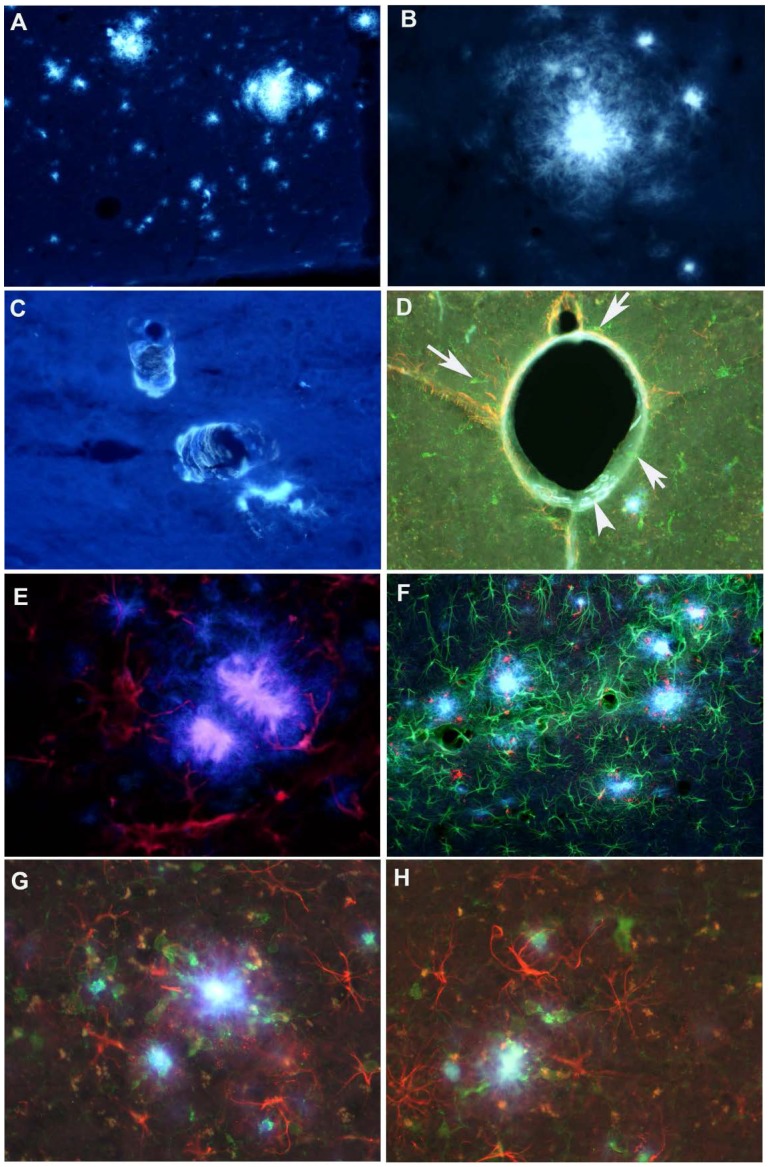
Medium (**A**) and high (**B**) power photomicrographs through the neocortex of the AD/Tg mouse brain showing A-G labeled amyloid plaques; (**C**) High power photomicrograph shows the vascular plaques stained with A-G; (**D**) Combined GFAP immunofluorescent staining (red) of activated astrocytes can be seen surrounding the A-G labeled vascular plaques (blue, arrow head), and activated microglia (green, arrows); (**E**) Combined GFAP immunofluorescent staining (red) of activated astrocytes around the A-G labeled plaques (blue); (**F**) Medium power photomicrographs reveals the distribution of A-G labeled plaques (blue) in association with hypertrophied astrocytes (green) and CD 68 activated microglia; red); (**G**,**H**) High power photomicrographs through the neocortex showing the activated astrocytes (red) and activated microglia (green) and A-G labeled (blue) plaques in the center.

Amylo-Glo labeling of parenchymal plaques is seen in the cortex of AD Tg (APPPS1) mice (Figure 4A,B). The vascular plaques are also brightly labeled with Amylo-Glo (Figure 4C,D). The activated astrocytes were found around the plaques in the cortex (Figure 4E). When multiple labeling was performed, activated microglia and hypertrophied astrocytes were observed around the plaques (Figure 4F–H).

## 15. Importance and Significance

Amylo-Glo stain has similar staining affinities for beta amyloid in the brain as the conventional markers Congo Red, Thioflavin S and Pan-A beta _40_ immunofluorescence. Hoever, Amylo-Glo exhibits a number of advantages including its compatibility with other histochemical procedures or immunohistochemical labeling. Also, the conditions for Amylo-Glo staining are very mild unlike other conventional markers such as Congo Red and Thioflavin S where harsh reagents are used. Another unique advantage of Amylo-Glo is its brightness enables one to quantify even at lower magnification, cutting down the time required for image analysis. 

## 16. Conclusions

While methodologies exist for labeling neuronal dysfunction, myelin pathology and glial cell activation, relatively few methods were developed to detect and localize brain vascular pathologies. To fulfill this need, the four fluorescent histochemical tracers described in this review were developed. Their specific functions are as follows:

1) Fluoro-Ruby, in addition to being a useful fluorescent antegrograde tract tracer, can be injected into the lateral ventricle of the brain to label all vascular pericytes under normal and pathological conditions;
2) Fluoro-Gold, in addition to being a useful fluorescent retrograde tract tracer, it can be injected I.P. to label all vascular endothelial cells in the normal or neurotoxicant exposed brain;
3) Fluoro-Turquoise conjugated gelatin can be administered via trans-cardiac perfusion to label the lumen of both normal and pathological blood vessels in the brain;
4) Amylo-Glo is specific for the fluorescent histochemical staining of amyloid plaques. As such, it will label both vascular and parenchymal plaques, which have very different pathologies.
